# National Physical Fitness and Sports Month — May 2013

**Published:** 2013-05-03

May is designated National Physical Fitness and Sports Month to raise awareness about the important role physical activity plays in maintaining health. According to the 2008 *Physical Activity Guidelines for Americans*, physical activity can help control weight, improve mental health, and lower the risk for early death, heart disease, type 2 diabetes, and some cancers. Physical activity also can improve cardiovascular and muscular fitness (1). In 2011, however, only one in five U.S. adults participated in enough physical activity to gain substantial health benefits (2).

To achieve substantial health benefits, the guidelines recommend that adults perform at least 150 minutes a week of moderate-intensity aerobic activity, or 75 minutes per week of vigorous-intensity aerobic activity, or an equivalent combination of moderate- and vigorous-intensity aerobic activities (1). The guidelines also recommend including muscle-strengthening activities that involve all major muscle groups on 2 or more days a week. Additional information about physical activity and resources for increasing participation in physical activity are available at http://www.health.gov/paguidelines and http://www.cdc.gov/physicalactivity.
